# Cardiac Arrest Induced by an Anaphylactic Reaction Associated With the First Dose of Cetuximab

**DOI:** 10.7759/cureus.26351

**Published:** 2022-06-26

**Authors:** Atsuya Hane, Asami Ito, Ken Ishikura, Hiroshi Imai, Yoshinaga Okugawa

**Affiliations:** 1 Emergency and Critical Care Center, Mie University Hospital, Mie, JPN; 2 Department of Genomic Medicine, Mie University Hospital, Mie, JPN

**Keywords:** cetuximab, anticancer drug, α-gal syndrome, cardiac arrest, anaphylaxis

## Abstract

Cetuximab is a chimeric mouse-human monoclonal antibody biologic used for the treatment of epidermal growth factor receptor-positive colorectal cancer and head and neck cancer. The incidence of severe anaphylaxis after infusion of cetuximab is a rare but fatal complication. Galactose-α-1,3-galactose (α-gal), a side-chain component in cetuximab, can cause the α-gal syndrome, an allergic cross-reaction to the α-gal contained in mammalian muscle. Here, we report a case of cardiac arrest induced by an anaphylactic reaction from cetuximab infusion. After the initial dosing of cetuximab in an outpatient setting, the patient developed sudden cardiac arrest. Flushing of the skin and bronchoconstriction led to the diagnosis of a severe anaphylactic reaction, whereupon he was treated with repeated doses of epinephrine, steroids, and continuous epinephrine infusion. The patient responded well to initial treatment, leading to a full recovery. The patient’s history and subsequent blood tests did not show any meat allergies. As an increasing number of patients receive chemotherapy as outpatients, it is important to be aware of the possibility of severe allergic reactions induced by these drugs.

## Introduction

Anaphylaxis is an allergic systemic reaction in which the human immune system responds to allergens and immune cell-released chemical mediators. Although rare, anaphylaxis can cause a fatal reaction and cardiac arrest, especially in the case of drug infusions, because the allergen is injected directly into the blood, with fatalities occurring in 0.7-2% of cases [1]. Cetuximab is a chimeric mouse-human monoclonal antibody used for the treatment of epidermal growth factor receptor (EGFR)-positive colorectal cancer and head and neck cancer [2]. A combination of cetuximab, encorafenib, and binimetinib was shown to result in a significantly longer overall survival and a higher response rate than standard therapy in patients with metastatic colorectal cancer with the *BRAF V600E* mutation who had had disease progression after one or two previous regimens [3]. The galactose-α-1,3-galactose (α-gal) syndrome is triggered by specific immunoglobulin E (IgE) directed against the immunogenic sugar α-gal, typically found in mammalian meat, milk, and products derived thereof, potentially resulting in anaphylaxis [4]. Here, we report a case of cardiac arrest caused by an anaphylactic reaction to an initial dose of cetuximab.

## Case presentation

**Figure 1:**
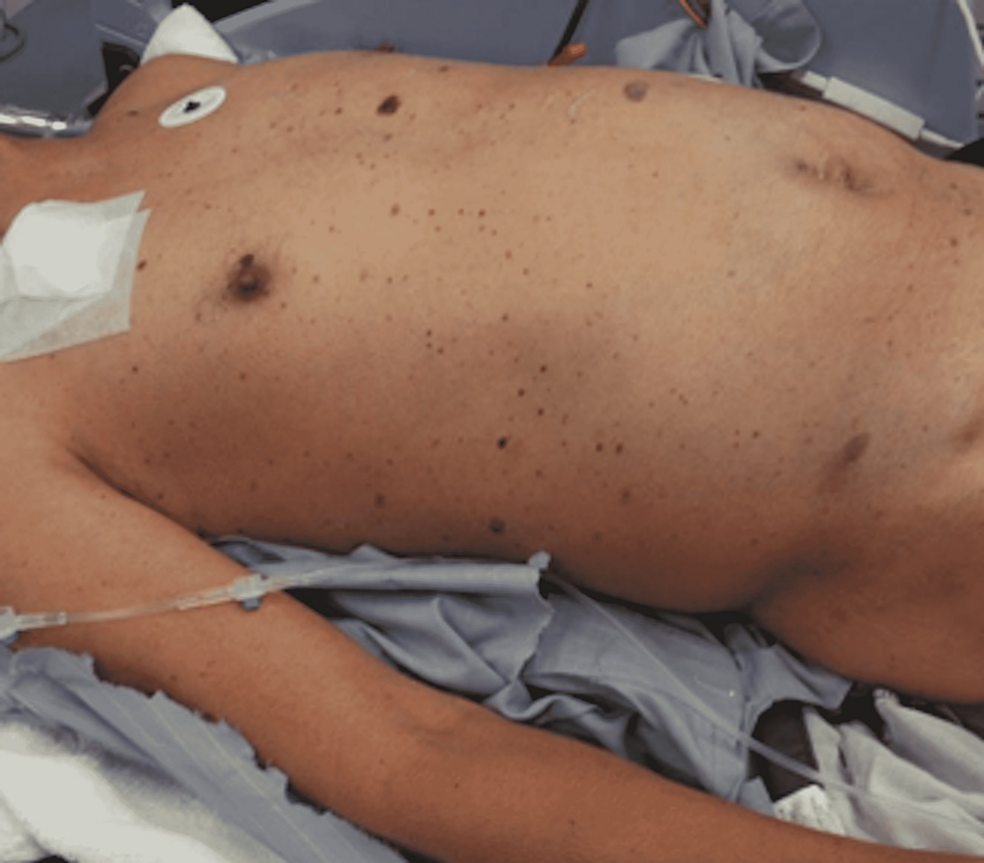
Skin rash on the patient’s trunk.

**Figure 2:**
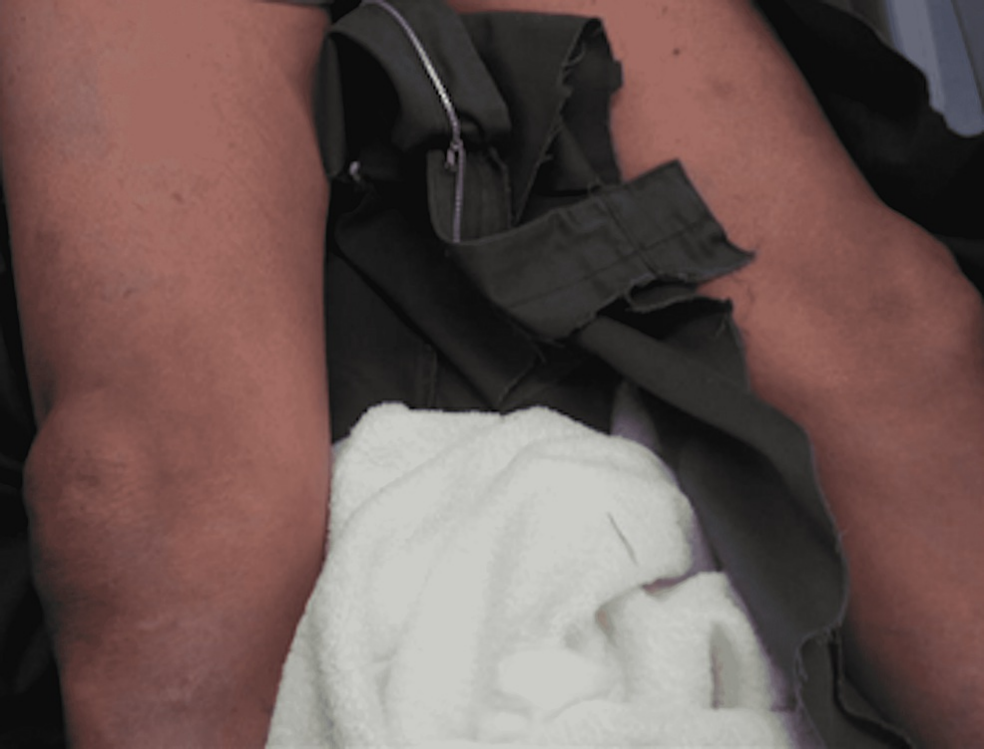
Skin rash on both lower legs of the patient.

A 72-year-old man after laparoscopic right hemicolectomy diagnosed with ascending colon cancer with multiple lung metastases visited the outpatient chemotherapy clinic for his first dose of cetuximab treatment as part of triplet therapy (combination of encorafenib, cetuximab, and binimetinib). He was receiving treatment for hypertension and hyperuricemia. A medical checkup conducted before the infusion showed no apparent abnormalities. Seven minutes after the infusion was initiated, his blood pressure was 81/48 mmHg, his facial skin showed flushing, and his consciousness was clear. However, eight minutes after starting the infusion, he went into sudden cardiac arrest. Medical staff performed cardiac resuscitation, administering 1 mg of epinephrine intravenously three times. When emergency physicians arrived, the monitor showed sinus brady. We performed tracheal intubation immediately but experienced high resistance upon ventilation with the self-inflating bag of a bag valve mask. The patient regained spontaneous circulation 12 minutes after cardiac arrest. We used a total of 3 mg of epinephrine. At that time, we noticed that his skin was flushing (Figures 1, 2). This, in addition to the high ventilatory effort indicating bronchospasm, suggested that the cardiac arrest had been due to an anaphylactic reaction. After achieving return of circulation, the patient was quick to regain consciousness; however, refractory hypotension persisted, requiring a continuous infusion of epinephrine for more than 12 hours. We used an additional 1,000 mg methylprednisolone intravenous injection in the emergency room. Bronchoconstriction was not apparent during ventilation management, so he did not warrant a high-pressure setting. He was extubated the next day, hospitalized in the intensive care unit for three days, and left the hospital three days later after achieving a full recovery. Subsequent allergen tests were performed for certain foods and natural substances, but the results showed normal levels of serum-specific IgE for pork, beef, and lamb (IgE for pork <0.10 [0.00-0.34], IgE for beef 0.23 [0.00-0.34], IgE for lamb 0.14 [0.00-0.34]). In addition, upon inquiry, the patient denied any history of food allergies. After discharge, the patient resumed treatment with another anticancer drug.

## Discussion

Anaphylaxis is an acute, life-threatening, multisystem syndrome caused by the sudden release of mast cell mediators into the systemic circulation. It most often results from IgE-mediated reactions to foods, drugs, and insect stings [5]. Severe anaphylaxis is a systemic reaction affecting ≥2 organs or systems and affects 1-3 per 10,000 people; however, in the United States and Australia, figures are even higher. Severe anaphylaxis is estimated to cause death in 0.65-2% of patients (i.e., 1-3 per million people) [1]. Drug injection-induced anaphylaxis has a rapid onset and can be quite severe because of the direct administration of the antigen into the blood. Sometimes it is difficult to diagnose such cases because of the presence of a mimic and the variable nature of the symptoms. The median time to respiratory or cardiac arrest is 30 minutes for foods, 15 minutes for venom, and five minutes for iatrogenic reactions [6]. The carbohydrate moiety α-gal is abundantly expressed on cells and tissues of all mammalian species except for primates, being especially abundant in meat (except for chicken) [7]. In addition, tick bites also appear to result in sensitization to α-gal [8]. The α-gal syndrome (AGS) is a type 1, IgE-mediated allergy to the oligosaccharide α-gal present in nonprimate mammals [9]. Patients with IgE to α-gal may show severe hypersensitivity reactions to α-gal-containing meats as well as the monoclonal antibody cetuximab [10]. Notably, the frequency of fatal anaphylaxis associated with the first dose of cetuximab is 0.8% [11]. Anaphylaxis induced by anticancer agents can be fatal. Such cases usually present with respiratory symptoms and a generalized skin rash, but in the present case, these symptoms appeared after cardiac arrest, making it difficult to diagnose anaphylaxis in the early stages. If we diagnose anaphylaxis earlier, we are able to intervene, such as by administering steroids. We found no literature concerning the time and course of fatal anaphylaxis leading to cardiac arrest after the first dose of cetuximab. Fatal anaphylaxis associated with drug administration is sometimes difficult to diagnose early because of its rapid course and atypical symptoms. Although antigen-specific anti-IgE antibodies may need to be measured to diagnose AGS, there have been reports of anaphylaxis occurring even when antibodies to meat products were negative [12]. In fact, in the present case, all meat-specific anti-IgE antibodies were negative. While it is possible that the present case was not one of AGS, it may be useful to check for a history of meat allergy or tick bite before the administration of drugs containing α-gal, such as cetuximab, to estimate the risk of anaphylaxis.

## Conclusions

Although our patient showed rapid progression to cardiac arrest, we were able to save his life with no sequelae, such as post-resuscitation encephalopathy. With the recent increase in the number of outpatients receiving chemotherapy, awareness of AGS, inquiring about a history of meat allergies prior to drug administration, and training in cardiopulmonary resuscitation will likely become increasingly important.
